# Supplementary material for: Evaluating Explainable Artificial Intelligence (XAI) techniques in chest radiology imaging through a human-centered Lens
Source: PLoS One. 2024 Oct 9;19(10):e0308758. doi: 10.1371/journal.pone.0308758 (PMC11463756; doi:10.1371/journal.pone.0308758)
Supplement: Attachment — Submitted filename: Response to Reviewers.pdf [file pone.0308758.s003.pdf]

Response to Reviewers

Dear Editor and Reviewers,

We sincerely appreciate the time and effort you and the reviewers have dedicated to evaluating our paper and offering valuable feedback. Your insightful comments have significantly contributed to the improvements in this revised version. We have carefully considered each suggestion and endeavoured to address them thoroughly. We hope the revised manuscript meets your high standards and we welcome any further constructive feedback.

Below, we provide our point-by-point responses. As requested, all modifications in the marked-up manuscript have been highlighted in yellow.

Reviewers' comments (highlighted in black text below) have been addressed in the updated manuscript and our response is provided in the blue text below.

Sincerely,

Shereen Fouad, PhD, SFHEA
s.fouad@aston.ac.uk
Senior Lecturer in Computer Science
Aston University (College of Engineering and Physical Sciences)

Journal Requirements

Requirement 1. When submitting your revision, we need you to address these additional requirements.

Please ensure that your manuscript meets PLOS ONE's style requirements, including those for file naming. The PLOS ONE style templates can be found at https://journals.plos.org/plosone/s/file?id=wjVg/PLOSOne_formatting_sample_main_body.pdf and https://journals.plos.org/plosone/s/file?id=ba62/PLOSOne_formatting_sample_title_authors_affiliations.pdf.

Response – Thank you for the kind reminder. The manuscript and file names have been carefully reviewed and updated to meet the PLOS ONE's style requirements.

Requirement 2. When submitting your revision, we need you to address these additional requirements.

Please note that PLOS ONE has specific guidelines on code sharing for submissions in which author-generated code underpins the findings in the manuscript. In these cases, all author-generated code must be made available without restrictions upon publication of the work. Please review our guidelines at https://journals.plos.org/plosone/s/materials-and-software-sharing#loc-sharing-code and ensure that your code is shared in a way that follows best practice and facilitates reproducibility and reuse.

Response – Thank you for the kind reminder. The author-generated an open access code on which the manuscript is based, has been provided as a supporting information

- S1 Supporting Information. Colaboratory Python code for clinical case study 1 - using Chest X-ray Images [32] - Explainable Deep Learning model for Pneumonia detection using Chest X-ray Images
- S2 Supporting Information, Colaboratory Python code for clinical case study 2 - using Chest CT Images [34] Explainable Deep Learning model for COVID-19 detection using Chest CT Images

The code is shred in a way that follows best practice and facilitates reproducibility and reuse.

Requirement 3. Your ethics statement should only appear in the Methods section of your manuscript. If your ethics statement is written in any section besides the Methods, please move it to the Methods section and delete it from any other section. Please ensure that your ethics statement is included in your manuscript, as the ethics statement entered into the online submission form will not be published alongside your manuscript

Response – Thank you for the kind reminder. Our proposed work consists of two main phases, presented in sections 3 and 4.

In phase one (section 3 Clinical case studies), we design and implement two clinical case studies using two open access datasets. The ethics statement for this part is now available in section 3.1 (Dataset Description, Pre-processing, and Ethics), and is not currently present in any other section.

In phase two (section 4 XAI human-centered evaluation method), we conducted a user study to evaluate their usefulness in the context of chest radiology imaging. The ethics statement for this part is now available in section 4.2 (User study ethical statement), and is not currently present in any other section.

Requirement 4. We note that Figure(s) 3 and 4 in your submission contain copyrighted images. All PLOS content is published under the Creative Commons Attribution License (CC BY 4.0), which means that the manuscript, images, and Supporting Information files will be freely available online, and any third party is permitted to access, download, copy, distribute, and use these materials in any way, even commercially, with proper attribution. For more information, see our copyright guidelines: http://journals.plos.org/plosone/s/licenses-and-copyright.

Response – Thank you for your comment. We have removed figures 3 and 4, and supplied replacements with other figures that convey the same information using other example images. The new figures (Fig 6 and 7) comply with the CC BY 4.0 license.

In particular, Figures 6 (a and e) display example unprocessed X-ray images - extracted from an open access datasets [Mendeley Data] (https://data.mendeley.com/datasets/rscbjbr9sj/2), licensed under CC BY 4.0 (https://creativecommons.org/licenses/by-nc-sa/4.0/), which allows for sharing, copying, and modifying the data for research purposes.

Figures 7 (a and e) display example unprocessed CT lung images - extracted from an open access datasets Kaggle SARS-CoV-2 CT-Scan Dataset](https://www.kaggle.com/datasets/plameneduardo/sarscov2-ctscan-dataset), licensed under CC BY-NC-SA 4.0, which allows for sharing, copying, and modifying the data for research purposes.

Both open-access datasets have been ethically approved by the data providers and the Aston University Ethical Approval Committee (approval number 234700-06).

The remaining subfigures (b,c,d,f,g,h) in both Figures 6 and 7, were exclusively produced as outputs of the code developed for this manuscript, making them unique to our study. All figures have been explained in the new figure captions – highlighted in yellow in the resubmitted manuscript).

The data sources and copyright licenses have been further clarified in section 3.1 (Dataset Description, Pre-processing, and Ethics).

Requirement 5. Kindly upload a separate folder for figure(s) 1 to 26. Please amend the file type to 'Figures'.

Response – Thank you for the kind reminder. The figures have been renamed appropriately and uploaded in a separate folder titled ‘Figures’

Additional Editor Comments: This manuscript has some merit, however it is not in a fine shape for consideration of acceptance in its current shape. Beside addressing the reviewers comments, the authors are asked to further motivate the chose of the xAI method used. In addition, the importance of xAI in every application domain (such as space [1] and intrusion detection [1]) should be discussed.
[1]https://doi.org/10.1016/j.engappai.2024.108517.
[2]https://doi.org/10.1016/j.eswa.2023.121751

Response – Thank you for the positive assessment of our manuscript, we appreciate your useful suggestions in improving the quality of our work and have made several modifications to ensure that the updated manuscript is up to the high standard of PLOS ONE publications.

Regarding the importance of XAI, we included a new text in section 1 (Introduction) (paragraph 2 - highlighted text) emphasising the importance of XAI in high-stakes domains, including space and cybersecurity, while referring to the suggested relevant articles [10,11].

Furthermore, we included a detailed discussion in section 3.3 (Visual Explainability models) justifying why Grad-CAM and LIME were the chosen xAI techniques in our study. The new text has been included in section 3.3 paragraph 1, and it is highlighted in yellow in the resubmitted manuscript.

Response to Reviewer 1

General Comment - The authors proposed XAI model to predict the status of COVID-19.It is DL-enabled diagnostic systems in chest radiography. Two prominent XAI methods, Grad-CAM and LIME, are employed to generate visual explanations of the AI decision-making process. Two clinical scenarios for diagnosing pneumonia and COVID-19 using DL techniques are evaluated, achieving accuracy rates of 90% for pneumonia and 98% for COVID-19. The model seems interesting and may gain many interests, However, I have minor suggestions:

Response – Thank you very much for your detailed review of our manuscript and your positive assessment. Your useful suggestions have been addressed below and included in our manuscript as advised.

Suggestion 1 - The authors msy highlight recent XAI-Covid models. I suggest to highlight PMID:36738712 and similar methods.

Response – Thank you for your suggestion, we highlighted recent XAI-Covid models by briefly discussing and citing the recommended article (reference 27) in section 2 (Literature review) paragraph 1, and the new text is highlighted in yellow in the updated manuscript.

Suggestion 2 - AUCROC plot must be drawn with mulitiple running points.

Response – Thank you for your suggestion, AUCROC plots have been provided for both datasets 1 and 2 (clinical case studies) in Figure 3 (3a and 3b), respectively. The figures are explained in section 3.2.2 paragraph 2, and the new text is highlighted in yellow in the updated manuscript.

Suggestion 3 - how the authors checked whether the model overfits or not.

Response – Thank you for your question. We implemented several techniques throughout our study to monitor and mitigate overfitting. In particular, our overfitting mitigation approach have been explained in the updated manuscript in section 3.2.1 (Experimental Settings) and the new text is highlighted in yellow in the updated manuscript.

We mentioned the followings: "*We implemented several techniques throughout our study to monitor and mitigate overfitting. This includes applying regularization techniques, specifically L2 regularization and dropout to penalize model complexity and minimizing the risk of overfitting. For instance, a dropout rate of $1^{-0.5}$ was used in the MobileNetV2 and DenseNet169 models (best performing) to classify chest X-ray images and CT scans into pneumonia and normal, and COVID-19 and Non-COVID-19 cases, respectively. We also utilised early stopping in the models to stop*

training the model after its optimal number of iterations has been reached. Furthermore, both the training and validation loss curves were continuously monitored to ensure that no significant divergence between these curves occurred, which is often a good indicator of overfitting."

Response to Reviewer 2

General Comment - The paper analyzes the usefulness of xAI techniques (particularly Grad-CAM and LIME) in chest radiology (X-ray and CT) through the lens of the medical professionals who would leverage such advancements in a clinical context. Such an approach is critical in evaluating xAI techniques.
The study suggests that Grad-CAM was preferred over LIME regarding coherency and trust, and medical professionals are not aware of the potential uses of xAI.
Overall, the paper clearly identifies the gap that it aims to address (human-centered evaluation of Grad-CAM and LIME on a real use case of CAD), uses a grounded approach, and extracts reasonable conclusions from the results.

Response – Thank you very much for your thorough review of our manuscript and your thoughtful evaluation. Your useful suggestions have been addressed below and included in our manuscript as advised.

Suggestion 1 - However, the paper could benefit from a more detailed explanation of why Grad-CAM and LIME were the chosen xAI techniques when there are many others available (for example, SHAP is even mentioned in the literature review).

Response – we included a detailed discussion in section (3.3 Visual Explainability models) justifying why Grad-CAM and LIME were the chosen xAI techniques in our study. and the new text is highlighted in yellow in the updated manuscript.

We explained that:

"Based on our initial experimental findings, Grad-CAM [12] and LIME [13] provide more stable and accurate localized explanations compared to SHAP [14] in both image classification tasks. Therefore, in this paper, we selected LIME and Grad-CAM methods due to their superior performance in delivering accurate, relevant, and stable explainability results. Evidence from recent literature supports this choice. For instance, a study in remote sensing image classification [35] compared the performance of ten different XAI methods and found that Grad-CAM and LIME were the most interpretable and reliable. Similarly, a research in [16] comparing Grad-CAM, SHAP, and LIME in the context of medical imaging concluded that Grad-CAM and LIME were more reliable, whereas SHAP was not the best for local accuracy in this application. This is consistent with findings in [14], which highlights that while SHAP provides comprehensive feature importance in non-imaging datasets, it may produce less stable explanations in complex image classification tasks, leading to potential

inconsistencies. These findings underscore the reliability and relevance of LIME and Grad-CAM in our study, facilitating better insights and trust in the model outputs. "

The above text was included in section 3.3 pages 6&7, and it is highlighted in yellow in the updated manuscript.

Suggestion 2 - It may also benefit from showing the performance of the discarded CNN architectures so one can better understand the weight of each evaluation metric in determining the model's overall performance. As of now the influence of each metric seems arbitrarily defined.

Response – Thank you for your suggestion, we have included the experimental results obtained from all the studied deep learning models for dataset 1 and 2 in Tables 1 and 2, respectively. The test results are reported in section 3.2.2 (Results). The Performance Metrics (on testset) of Deep Learning Models on Dataset 1 and 2 are reported in terms of Accuracy, Precision, Recall, and F1 Score. We also report the training and validation loss and accuracy plots for the best performing models, in Dataset 1 and 2, in Figures 1 and 2, respectively. The updated text is highlighted in yellow in the updated manuscript.
